# Infants with retinopathy of prematurity (ROP) in Germany and Türkiye–a comparison based on data from the EU-ROP registry

**DOI:** 10.3389/fmed.2026.1868323

**Published:** 2026-07-29

**Authors:** Johanna M. Pfeil, Sophie C. Schnorr, Emely Meinheit, Şengül Özdek, Aslihan Uzun, Caner Kara, Teresa Barth, Ozlem Eski Yucel, Ameli Gabel-Pfisterer, Verena Schöneberger, Sibel Kadayifcilar, Tim Bleul, Helge Breuss, Huban Atilla, Tim U. Krohne, Karsten Hufendiek, Nicole Eter, Sabine Aisenbrey, Christos Skevas, Daniela Süsskind, Konrad Schulze, Püren Işık, Waldemar Jendritza, Stefanie Gniesmer, Marlene Saßmannshausen, Georg Spital, Vinodh Kakkassery, Tarek Aljarmakani, Imren Akkoyun, Iva-Ruth Bartmann, Sabine Baumgarten, Robert P. Finger, Melih Parlak, Erik Chankiewitz, Andreas Stahl, EU-ROP Study Group, Jakob Briem, Johann Colvin, Stefan Egger, Herbert Reitsamer, Patrick Schernthaner, Sandra Stuppner, Eva Wiener, Lilyana Petrova Dimitrova, Iliyana Georgieva Ilieva, Gergana Svetoslavova, Ognyan Mitkov Mladenov, Alexander Hugo Oscar, Yanitsa Kondova-Atanassova, Vasil Marinov, Mirjana Bjelos Roncevic, Jelena Doresic, Edita Kondza Krstonijevic, Rebeka Ribičić, Jiří Dušek, David Honer, Michaela Štěpanyová, Barbora Vařejková, Piret Jüri, Liina Kala, Mikk Pauklin, Amandine Barjol, Georges Caputo, Fiammetta Catania, Florence Metge-Galatoire, L. Geerdts, Madlen Löbel, Lisa Starke, Erik Chankiewitz, Spyridon Monastiriotis, Georgia Potaki, Florian Gekeler, René Machulla, Marius Metz, Ameli Gabel-Pfisterer, Thomas Pohlenz, Julia M, Pulst Caliman, Sophie Korn, Sandra Kroll, Martina Möglich, Helge Breuß, Anne-Kathrin Retzlaff, Waldemar Jendritza, Bettina Jendritza, Zakaria Drebi, Florian Schindler, Henrik Faatz, Leonie Kläver, Andrea Koschinski, Denise Ruch, Georg Spital, Michael Petrak, Kristina Pfau, Marlene Saßmannshausen, Annika Ader, Katrin Engelmann, Ulrike Fleischer, Imke Goldammer, Vinodh Kakkassery, Grabiela Krestanova, Franziska Mühmel, Pavel Smetana, Martin Tiez, Anja Wienigk, Jemina Benga, Rainer Guthoff, Stephan Jansen, Sema Kaya, Ala Khamees, Jennifer Prues-Hölscher, Alexandra Schilcher, Verena Schöneberg, Alexander Bartsch, Lisa Dussing, Sabrina Frank, Karolina Motloch, Eva Biewald, Tobias Kiefer, Mael Lever, Philipp Rating, Tim Bleul, Alexandra T. Camp, Navid Farassat, Nikolai Gross, Lutz Joachimsen, Sebastian Küchlin, Wolf A. Lagrèze, Luca Malagutti, Fanni E. Molnár, Michael Reich, Anne Schwietering, Iva-Ruth Bartmann, Fithri Indraswari, Monika Andrassi-Darida, Kerstin Holve, Kafa Ilia, Lyubomyr Lytvynchuk, Tarek Aljarmakani, Sebastian Bemme, Jana Katharina Dieks, Mohammed Khattab, Peer Lauermann, Yulia Meyer, Hagen Bahlmann, Matthias Heckmann, Knud Linnemann, Konrad Schulze, Andreas Stahl, Allam Tayar, Hanna Faber, Nefeli Kounatidou, Caroline Lago, Laurenz Johannes Bernhard Pauleikhoff, Tim Schade, Christos Skevas, Martin Spitzer, Annabelle Volk, Jan Wildner, Denise Yang-Seeger, Anna Bajor, Anke Beckmann, Carolin Böhne, Bettina Bohnhorst, Dorothee Brockmann, Meltem Elcivan, Carsten Framme, Thomas Gröber, Melanie Haar, Jonas Herden, Karsten Hufendiek, Christoph Jacobi, Christina Jacobsen, Nils Mester, Corinna Peter, Amelie Pielen, Sabine Pirr, Tjark Rauscher, Alena Riesmeier, Lorena Stachowski, Nora Wojtera, Aida Shaghaghi Zadeh, Tim U. Krohne, Jeany Li, Nour Arbaoui, Sabine Buchner, Felix Dittgen, Elke Edelmann, Robert P. Finger, Mustafa Kamal Hallak, F. Kipfmüller, Sina Koch, Jonathan Schaeff, Sylvia Selig, Verena Englmaier, Nicole Eter, Silvia Falkenau, Barbara Glitz, Sarah Kleemann, Camila Matias, Jenny Potratz, Julia Sandkötter, Jens Storp, Maria Tekaat, Cedric Weich, Teresa Barth, Lauretta Gluth, Horst Helbig, Herbert Jägle Annette Keller-Wackerbauer, Florian Langhammer, Holger Michel, Sven Wellmann, Steffi Knappe, Sabine Baumgarten, Volker Buck, Emilia Michalewicz, Peter Walter, Tibor Lohmann, Viktorija Belousova, Maximilian Busch, Stefanie Gniesmer, Salvatore Grisanti, Julius Caspar Rotering, Christian Schiemenz, Philip Schubart, Axel Frank, Ulrike Hagemann, Benedikt Rössler, Daniela Süsskind, Michael Völker, Britta Dahlke, Melih Parlak, Ingrid Weithmann, Armin Wolf, Johannes Kahle, Wessam Alkabouni, Sabine Aisenbrey, Lisa Fu, Marian Liegl, Melanie Michelczak, Maria Lithoxopoulou, Asimina Mataftsi, Stella Moutzouri, Christos Tsakalidis, Nikolaos Ziakas, Theodoros Gkouvas, Tataropoulou Kassandra, Agathi Kouri, Anna Mourgela, Angeliki Nika, Margarita Papadopoulou, Sofia Androudi, Maria Goudesidou, Ioanna Grivea, Katerina Kaffe, Eleni Papageorgiou, Zoi Tsani, Teodóra Dusek, Erika Maka, Miklós Szabó, Andrea Szigeti, Lilána Szücs-Székács, Mediha Bahtiri, Fjolla Bokshi, Shukrije Brajshori, Florie Greicevci, Luan Morina, Belinda Pustina, Lisa Qurdina, Ardian Shabani, Mazur Liubovi, Maria Szwajkowska, Kristof Cieslik, Agnieszka Czeszyk, Dariusz Gruszfeld, Wojciech Hautz, Beata Kocyla-Karczmarewicz, Klaudia Rakusiewicz-Krasnodȩbska, Anna Rogowska, Monika Oziȩbło-Kupczyk, Joanna Zawistowska, Magdalena Kaszuba-Modrzejewska, Bogumiła Wójcik-Niklewska, Elżbieta Czapla, Julia Dezor-Garus, Anna Gotz-Wiȩckowska, Jolanta Kaczmarek, Jan Mazela, Marta Pawlak, Tomasz Szczapa, Dawid Szpecht, Nicoleta Grosu, Mădălina Hapca, Monica Hăşmăşanu, Melinda Matyas, Simona Nicoară, Claudia ştefănuţ, Gabriela Zaharie, Julia Escudero Gómez, Lorena Azorín-Pérez, Honorio Barranco-González, Carlos Cauto-Picazo, Isabel Pascual-Camps, Marta Solaz-Ruiz, Ester Torres-Martínez, Franscisca Barcos Munoz, Walid Bouthour, Eline De Clerck, Gunda Kann, Mateusz Kecik, Martina Kropp, Ariane Malclès, Gabriele Thumann, Tin Vo, Josep Callizo Planas, Daniela Hauenstein, Jordan Löliger, Huban Atilla, Pınar Bingöl Kızıltunç, Feyza Çalış Karanfil, Emel Okulu, Bensu Sezer, Imren Akkoyun, Ayse Ecevit, Gülsah Gökgöz, Deniz Anuk Ince, Gursel Yilmaz, Puren İsik, Hacer Yapicioglu Yildizdas, Cagatay Karaca, Mehmet Mutlu, Osman Ahmet Polat, Huseyin Pur, Ahmet Yağmur Baş, Caner Kara, Ayşen Sumru Kavurt, Betül Ortatatlı, İbrahim Murat Hirfanoğlu, Benay Karabulut, Esin Koç, Şengül Özdek, Hüseyin Baran Özdemir, Figen Bezci Aygün, Tolga Çelik, İrem İyigün, Sibel Kadayıfçılar, Ozlem Eski Yucel, Aslihan Uzun, Nilgün Köksal, Meral Yıldız, Ilke Mungan Akin, Tetiana Havrylyshyn, Paul Runge, Sergiy Lupyr, Serdeha Olha Oleksandrivna, Katsan Sergii Volodymyrovych

**Affiliations:** University Hospital Salzburg; Acibadem City Clinic Tokuda University Hospital EAD; University Hospital Alexandrovska; University Hospital Saint George, University Eye clinic and Medical University; Clinical Hospital Sveti Duh; České Budějovice; Eye Clinic of Tartu University Hospital; Hospital Rothschild Paris; Carl-Thiem University Hospital Cottbus; City Hospital Braunschweig; Clinic Stuttgart Location Katharinen hospital, part of Olga Hospital; Ernst von Bergmann Hospital Potsdam; Helios Hospital Berlin-Buch; Jendritza Ophthalmic Practice, Ludwigshafen; Nürnberg Clinic South; St. Franziskus Hospital Münster; University Hospital Bonn; University Hospital Chemnitz; University Hospital Düsseldorf; University Hospital Erlangen; University Hospital Essen; University Hospital Freiburg; University Hospital Fulda; University Hospital Gießen; University Hospital Göttingen; University Hospital Greifswald; University Hospital Hamburg-Eppendorf; University Hospital Hannover; University Hospital Köln; University Hospital Mannheim; University Hospital Münster; University Hospital Regensburg; University Hospital Rostock; University Hospital RWTH Aachen; University Hospital Schleswig-Holstein; University Hospital Tübingen; University Hospital Ulm; Vivantes Hospital Neukölln; Papageorgiou General Hospital of Thessaloniki, Aristotle University of Thessaloniki; P&A Kyriakou Athens Children Hospital; University Hospital of Larissa; Semmelweis University; University Clinical Center of Kosovo; Gheorghe Paladi Hospital; Prof. Dr. Stanislaw Popowski Regional Specialized Children’s Hospital; Children’s Memorial Health Institution; Medical University of Bialystok Children’s Clinical Hospital; University Hospital in Bydgoszcz; University Hospital in Zabrze Silesia; University of Medical Sciences Poznan; Cluj Country clinical emergency hospital; Biomédica de Málaga y Plataforma en Nanomedicina; University and Polytechnic Hospital La Fe; Service d’ophtalmologie University Hospital, Geneva; University Hospital Basel; Ankara University; Baskent University; Cukurova University; Erciyes University, Faculty of Medicine; Etlik Zübeyde Hanim Women’s Health Education and Research Hospital; Gazi University; Hacettepe University Children’s Hospital; Ondokuz Mayis University; Ordu University Medical Faculty; Uludag University Faculty of Medicine; Umraniye Research and Education; Ivano-Frankivsk Oblast Dytyacha Klinichna Likarnya; Kiev pediatric Hospital Ohmatdyt; The Filatov Institute of Eye Diseases and Tissue Therapy of NAMS of Ukraine; 1 Department of Ophthalmology, University Medicine Greifswald, Greifswald, Germany; 2 Department of Ophthalmology, Gazi University School of Medicine, Ankara, Türkiye; 3 Department of Ophthalmology, Ordu University Faculty of Medicine, Ordu, Türkiye; 4 Department of Ophthalmology, Etlik City Hospital, Ankara, Türkiye; 5 Department of Ophthalmology, University of Regensburg, Regensburg, Germany; 6 Department of Ophthalmology, Ondokuz Mayis University Faculty of Medicine, Samsun, Türkiye; 7 Department of Ophthalmology, Ernst-Von-Bergmann Hospital, Potsdam, Germany; 8 Department of Ophthalmology, HMU Health and Medical University Potsdam, Potsdam, Germany; 9 Department of Ophthalmology, Medical Faculty and University Hospital Düsseldorf, Heinrich Heine University Düsseldorf, Düsseldorf, Germany; 10 Department of Ophthalmology, Hacettepe University School of Medicine, Ankara, Türkiye; 11 Eye Center, Medical Center - Faculty of Medicine, University of Freiburg, Freiburg, Germany; 12 Department of Ophthalmology, Helios Hospital Berlin-Buch, Berlin, Germany; 13 Department of Ophthalmology, Ankara University Faculty of Medicine, Ankara, Türkiye; 14 Department of Ophthalmology, Faculty of Medicine and University Hospital Cologne, University of Cologne, Cologne, Germany; 15 University Eye Hospital, Hannover Medical School, Hannover, Germany; 16 Department of Ophthalmology, University of Muenster Medical Center, Muenster, Germany; 17 Department of Ophthalmology, Vivantes Klinikum Neukoelln, Berlin, Germany; 18 Department of Ophthalmology, University Medical Center Hamburg-Eppendorf, Hamburg, Germany; 19 University Eye Hospital, Eberhard Karls University of Tübingen, Tübingen, Germany; 20 Department of Ophthalmology, Çukurova University, Adana, Türkiye; 21 Jendritza Ophthalmic Practice, Ludwigshafen, Germany; 22 Department of Ophthalmology, University of Luebeck, Luebeck, Germany; 23 Department of Ophthalmology, University Medicine Bonn, Bonn, Germany; 24 Department of Ophthalmology, St. Franziskus-Hospital Muenster, Muenster, Germany; 25 Department of Ophthalmology, Klinikum Chemnitz gGmbH, Chemnitz, Germany; 26 Department of Ophthalmology, University Medical Center Goettingen, Goettingen, Germany; 27 Department of Ophthalmology, Baskent University Faculty of Medicine, Ankara, Türkiye; 28 Department of Ophthalmology, Klinikum Fulda, Faculty of Medicine/Campus Fulda, University of Marburg, Fulda, Germany; 29 Department of Ophthalmology, University Hospital RWTH Aachen, Aachen, Germany; 30 Department of Ophthalmology, Medical Faculty Mannheim of the Ruprecht-Karls-University Heidelberg, Mannheim, Germany; 31 Department of Ophthalmology, Ulm University Hospital, Ulm, Germany; 32 Department of Ophthalmology, Academic Hospital Braunschweig, Braunschweig, Germany

**Keywords:** anti-VEGF, Germany, laser, registry, retinopathy of prematurity, Türkiye

## Abstract

**Purpose:**

Data on regional differences in demographics and treatment patterns for treatment-warranted retinopathy of prematurity (ROP) is limited. In this study, data from the two countries with the highest patient numbers in the EU-ROP registry, Germany and Türkiye, are analyzed.

**Methods:**

The non-interventional, multicenter European ROP registry (EU-ROP) was established in 2021 to compare various parameters related to ROP. In this evaluation, 267 treated children (516 eyes) from Germany and 198 treated children (378 eyes) from Türkiye were included. The two cohorts were compared with regard to demographics, ROP treatment parameters and retreatment rates.

**Results:**

Infants in the German cohort had significantly lower gestational age (GA) as well as postmenstrual age (PMA) at initial treatment and (where applicable) at the time of retreatment. In the German cohort, the majority of eyes (56.1%) were treated due to stage 3 ROP in zone II with plus disease (II 3+). In the Turkish cohort, ROP II 3+ (22.0%), aggressive ROP (A-ROP) (17.9%), and ROP II 3- (17.3%) were the most common treatment indications. While ranibizumab was the predominant treatment modality in the German cohort (91.3%), about half of the eyes in the Turkish cohort were treated with laser coagulation (48.2%) while about 40% received intravitreal bevacizumab. Retreatment rates seemed to be comparable between Germany and Türkiye (28.1% vs. 27.3% of eyes) and were depending on the initial treatment method (30.5% after ranibizumab, 36.9% after bevacizumab, 14.2% after laser coagulation), although the retreatment indications [persistent avascular retina (PAR) vs. active ROP] appear to differ significantly between the two cohorts.

**Conclusion:**

In the investigated cohorts, ROP demographics, severity and treatment patterns differed significantly between Germany and Türkiye while the retreatment rate seems comparable. Different approaches to treatment of PAR may have contributed to the comparable retreatment rates despite the differences in initial treatment.

## Introduction

More than 80 years after the first description by Terry et al. (1) retinopathy of prematurity (ROP) is still a disease that can lead to severe visual impairment and even blindness (2). In recent years, several prospective randomized controlled trials (RCTs) have been conducted in this field, leading to significant changes in treatment patterns (3). As a result of these trials, two anti-VEGF drugs, ranibizumab and aflibercept, have been approved by the EMA for the treatment of ROP (ranibizumab at the end of 2019, aflibercept at the end of 2022) (4, 5). However, little is known about how the results of these clinical trials and the approvals of the two drugs translate into real-world clinical practice. In order to generate data that is representative beyond the boundaries of clinical trials or a single country, the European registry for retinopathy of prematurity (EU-ROP) was launched in 2021 to collect real-world ROP data in a multinational approach across geographical Europe.

This analysis represents the first comparison of the two countries with the highest patient numbers entered into the EU-ROP database, Germany and Türkiye. For all such comparisons, it should be remembered that ROP is a disease that is highly sensitive to subtle differences in neonatal and ophthalmological care (2, 6). One parameter capturing differences in neonatal care is the neonatal mortality rate per 1,000 live births which is published annually by the World Health Organization (WHO). Based on the latest WHO report from 2023, this parameter shows with 4.9 in Türkiye and 2.3 in Germany a more than 2-fold difference between the two countries (7). A direct comparison of ROP data from Germany and Türkiye has never been done before and is the focus of this study.

## Materials and methods

The EU-ROP registry is a non-interventional, multicenter, international registry that was established in 2021 as the European expansion of the German Retina.net ROP registry, which had been conducted between 2011 and 2020 (8, 9, 10). The registry is open to centers treating patients with ROP across geographical Europe. Participating centers consecutively document routine clinical data of infants who undergo treatment for ROP. The registry does not include all preterm infants or all infants diagnosed with ROP, but exclusively infants receiving any form of ROP treatment in routine clinical care, irrespective of the treatment modality. Eligible for documentation are all infants receiving treatment for ROP at participating centers and for whom informed consent for data collection and analysis has been obtained by parents or legal guardians. Both prospective and retrospective data entry are permitted provided written informed consent is available. Retrospective data entry explains why some data may date back to dates prior 2021. The study was approved by the leading Ethics committee in Greifswald (BB 165/19a) and at all local sites. The study is registered under DRKS00025138 at DRKS and under NCT04939571 at clinicaltrials.gov.

For the present study, all registry patients from Germany and Türkiye that were entered up to 7 January 2026 were screened for inclusion in the analysis. A total of 607 patients were identified (315 from Germany and 292 from Türkiye). Patients with unclear pre-treatment status (*n* = 39), previous ROP treatment at another hospital (*n* = 64), initial treatment before 2019 or after July 2025 (*n* = 38), or insufficient treatment documentation (*n* = 1) were excluded, resulting in 465 evaluable patients (Supplementary Figure 1). Patients whose initial treatment took place before 2019 were excluded to ensure a homogeneous study population as only few patients had been treated before 2019 and clinical practice changed considerably during this period. Patients whose initial treatment took place after July 2025 (i.e. less than 6 months before data export for the current analysis) were excluded to allow centers to document potential retreatments that may have become necessary. From all patients available for analysis, 267 patients (516 eyes) had been treated at 24 German centers and 198 patients (378 eyes) at eight Turkish centers. The registry does not define a birth-year eligibility criterion. In the present analysis, all but one child were born between June 2018 and May 2025; one child born in 2016 underwent treatment in 2024 because of persistent avascular retina (PAR) secondary to ROP and was thus entered in the registry in 2024 when this treatment occurred.

In the current analysis, we compare basic demographic parameters, clinical characteristics at initial ROP treatment, initial treatment parameters and retreatment parameters. Detailed information on which parameters are documented in the EU-ROP database has been published by Catt et al. (11) ROP severity has been diagnosed and documented in accordance with the current international classification for ROP (ICROP3) (12).

Since this is an observational registry, data are not always available for all patients/eyes, although sites are of course advised to enter data as completely as possible. To account for this fact, N indicates the number of patients or eyes in all analyses. Due to the fact that outliers, confirmed by the sites, were present in almost all parameters, Mann-Whitney-U tests were used for comparisons of groups and parameters were reported as median with interquartile range (IQR). Outliers in continuous variables were identified with the ROUT method (Q = 1%) (13) and were queried in the EU-ROP database. Only confirmed values were included in this analysis. The chi-square test and Fisher’s exact test were used to compare nominally scaled variables depending on group size. Treatment of zone III 2+, zone II 1-, zone III 1+, zone III 3-, zone III 2-, zone III 1-, and of eyes with incomplete vascularization or fully vascularized eyes at time of initial treatment (independent of type of treatment) were queried in the EU-ROP database. All parameters with open queries at time of data analysis were excluded from the current analysis. At time of retreatment, ROP status was only queried if anti-VEGF treatment was documented while retreatment with laser was assumed to be for PAR for the above listed ROP stages. A *p*-value of ≤0.05 was considered statistically significant and significant differences are indicated by stars (*≤0.05, **≤0.01, ***≤0.001). SPSS V.31 (IBM Corp., Armonk, NY, United States) and GraphPad Prism V.9.5.1 (GraphPad Software, Inc., La Jolla, California, United States) were used for statistical analyses.

## Results

### Demographic parameters

We observed statistically significant differences between the German and the Turkish cohort in all demographic parameters at birth, initial treatment, and first retreatment, apart from distribution of sex and plurality, and weight gain per day until treatment (Table 1).

**TABLE 1:** Demographic characteristics at birth, initial treatment, and first retreatment by country.

Demographic parameters		Germany	Türkiye	All patients	*P*-value
At birth	GA at birth (median in weeks) (IQR) (Germany *N* = 265; Türkiye *N* = 198)	24.7 (23.9–26.0)	28.2 (26.3–30.9)	25.7 (24.4–28.4)	**<0.0001**
	Birth weight (median in g) (IQR) (Germany *N* = 257; Türkiye *N* = 198)	600 (490–722)	1,005 (780–1,460)	720 (570–1,000)	**<0.0001**
	Sex, male (%) (Germany *N* = 267; Türkiye *N* = 198)	57.3	58.6	57.8	0.8178
	Plurality: singletons/twins/triplets (%) (Germany *N* = 267; Türkiye *N* = 198)	72.3/24.0/2.6	72.2/26.3/1.5	72.3/24.9/2.2	0.6469
At initial treatment	PMA (median in weeks) (IQR) (Germany *N* = 262; Türkiye *N* = 180)	36.7 (35.1–39.2)	38.7 (36.8–42.1)	37.6 (35.7–40.4)	**<0.0001**
	PNA (median in weeks) (IQR) (Germany *N* = 263; Türkiye *N* = 182)	11.7 (10.3–14.1)	10.8 (7.7–14.2)	11.4 (9.4–14.1)	**0.0017**
	Weight (median in g) (IQR) (Germany *N* = 132; Türkiye *N* = 154)	2,140 (1,601–2,565)	2,500 (1,988–3,453)	2,293 (1,749–2,900)	**<0.0001**
	Weight gain per day (mean in g) (SD) (Germany *N* = 132; Türkiye *N* = 153)	16.4 (14.0–16.4)	18.2 (12.9–23.2)	17.1 (13.8–17.1)	0.1096
At first retreatment	PMA (median in weeks) (IQR) (Germany *N* = 85; Türkiye *N* = 61)	42.0 (39.1–44.1)	47.1 (42.2–72.5)	43.1 (40.1–48.6)	**<0.0001**
	PNA (median in weeks) (IQR) (Germany *N* = 87; Türkiye N = 61)	17.6 (14.1–20.0)	21.4 (14.4–45.2)	18.5 (14.3–23.5)	**0.0011**
	Weight (median in g) (IQR) (Germany *N* = 46; Türkiye *N* = 25)	2,650 (2,200–3,500)	3,280 (2,690–5,000)	2,940 (2,300–3,650)	**0.0082**
	Time between initial treatment and first retreament (median in days) (IQR) (Germany *N* = 86; Türkiye *N* = 59)	48.5 (26.0–64.3)	56.0 (21.0–225.0)	49.0 (21.0–76.0)	**0.0345**

Bold values indicate the significant *p*-values.

For distribution of documented comorbidities in the two countries see Table 2. In most parameters the two countries were comparable, but a significant difference was observed regarding the distribution of necrotizing enterocolitis (NEC), which was documented with about equal prevalence in the two countries (18.9% vs. 19.9%) but with considerably more NEC surgeries in Germany (12.4%) than in Türkiye (5.0%). Oxygen supplementation and the use of mechanical ventilation in infants who received oxygen supplementation was comparable between the two cohorts - more than 90% of infants treated for ROP in both groups had received oxygen supplementation for more than 5 days before first ROP treatment (see Table 2).

**TABLE 2:** Comorbidities as documented in the EU-ROP registry by country.

Comorbidities documented in the EU-ROP registry	Categories	Germany (*N* = 267)	Türkiye (*N* = 198)	All patients (*N* = 465)	*P*-value
O_2_ supplementation *N*, (%)	None	3 (1.3)	3 (1.4)	6 (1.5)	0.4935^a^
	≤5 days	7 (3.1)	10 (5.4)	17 (4.1)	
	>5 days	216 (95.6)	173 (93.0)	389 (94.8)	
	Unknown	27	5	22	
	Missing	14	7	29	
Use of mechanical ventilation for O_2_ supplementation *N*, (%)	No	5 (2.2)	5 (2.9)	10 (3.2)	0.7528^b^
	Yes	215 (97.7)	165 (97.1)	300 (96.8)	
	Unknown	3	13	16	
	Missing	–	–	–	
Germ positive sepsis *N*, (%)	Absent	136 (62.7)	99 (55.0)	235 (59.2)	0.1254^b^
	Present	81 (37.3)	81 (45.0)	162 (40.8)	
	Unknown	30	13	43	
	Missing	20	5	25	
Intraventricular hemorrhage (IVH) *N*, (%)	Absent	141 (66.2)	129 (69.7)	270 (69.6)	0.1640^a^
	Grade I	14 (6.6)	19 (10.3)	33 (8.5)	
	Grade II	16 (7.5)	16 (8.4)	32 (8.2)	
	Grade III	27 (12.7)	10 (5.4)	37 (9.5)	
	Grade IV	9 (4.2)	6 (3.2)	15 (3.9)	
	Yes, grade unknown	6 (2.8)	5 (2.7)	11 (2.8)	
	Unknown	34	9	43	
	Missing	20	4	24	
Necrotising enterocolitis (NEC) *N*, (%)	Absent	176 (81.1)	145 (80.1)	321 (80.7)	**0.0015^a^**
	Without need for surgery	14 (6.5)	27 (14.9)	41 (10.3)	
	With need for surgery	27 (12.4)	9 (5.0)	36 (9.0)	
	Unknown	30	13	43	
	Missing	20	4	24	
Periventricular leucomalacia *N*, (%)	Absent	187 (92.1)	151 (84.8)	338 (88.7)	**0.0340^b^** –
	Present	16 (7.9)	27 (15.2)	43 (11.3)	
	Unknown	44	16	45	
	Missing	20	4	26	
Porencephaly *N*, (%)	Absent	197 (97.0)	179 (96.8)	376 (96.9)	>0.9999^b^ –
	Present	6 (3.0)	6 (3.2)	12 (3.1)	
	Unknown	44	9	39	
	Missing	20	4	26	

*^a^*Chi-square test.

*^b^*Fisher’s exact test was used to calculate *p*-value. Categories “unknown” and “missing” were excluded from these calculations. Bold values indicate the significant *p*-values.

In Germany, 11 of 267 infants (4.1%) died during the study period, at a mean of 36 days after the last ROP treatment (available for five infants). Most had initially received ranibizumab treatment, and five infants had undergone retreatment. Documented causes of death included pulmonary decompensation, multiorgan failure, bronchopulmonary dysplasia, and respiratory arrest.

In Türkiye, 6 of 158 infants (3.8%) died at a mean of 40 days after ROP treatment (available for three infants). Initial treatment had consisted of either bilateral laser or bilateral bevacizumab, and no infant had required retreatment. Documented causes of death were related to severe cardiopulmonary complications.

### ROP severity at initial treatment

**FIGURE 1:**
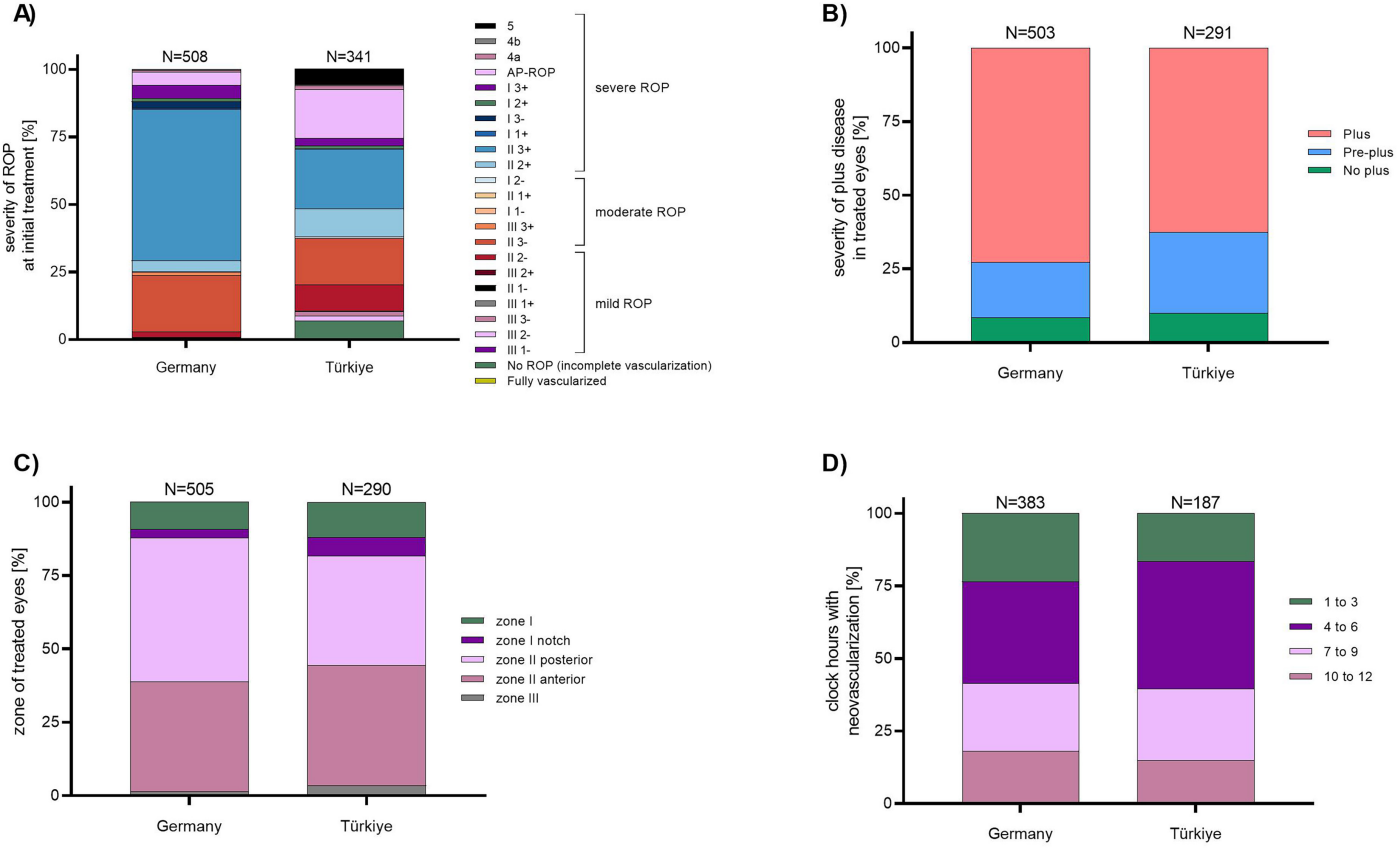
Severity of ROP at time of initial treatment. **(A)** ROP severity according to mROP-ActS by Pivodic et al. (13); **(B)** severity of (pre-)plus disease; **(C)** location of ROP by zones; **(D)** extension of neovascularizations in clock hours.

The most common ROP severity at initial treatment in the German cohort was zone II 3+, which was present in 56.1% of all eyes, followed by zone II 3- in 20.9%. Only 4.9% of infants in Germany were graded as aggressive ROP (A-ROP) at initial treatment and only very few had partial retinal detachment (4a: 0.6%, 4b: 0.4%). Stage 5 was not present in the German cohort at time of initial treatment. In the Turkish cohort, we observed more variability in the ROP grades at initial treatment. The most prevalent severity was also zone II 3+ (22.0%), followed by A-ROP in 17.9% of eyes and zone II 3- (17.3%). Stage 4a was present in 1.5%, 4b in 0.3% and stage 5 in 5.9% of eyes at initial treatment in Türkiye. Another interesting difference between the two countries is that in Türkiye 6.7% of all eyes had their first ROP treatment due to incomplete vascularization of the retina, while this was not observed in Germany (Figure 1A and Supplementary Table 1). It is important to note that pre-plus was counted as no plus disease when assigning severity stages according to the modified ROP activity score as proposed by Pivodic et al. (14)

In the German cohort, about three quarters of treated eyes had plus disease (72.8%), and 18.7% pre-plus disease. In the Turkish cohort about two thirds of eyes (62.5%) had plus disease and 27.5% pre-plus disease at time of initial treatment. In both countries, only very few eyes were treated without plus disease or pre-plus disease being present (Germany 8.6%; Türkiye 10.0%) (Figure 1B).

In Germany, zone II posterior was the most frequently affected zone at time of initial treatment (48.9%), followed by zone II anterior (37.4%). In Türkiye, zone II posterior and anterior were about equally prevalent (II posterior: 37.2%; II anterior: 41.0%). Zone I was present in Germany in only 9.3%, in Türkiye in 12.1%, and zone I secondary to notch was present in 3.0% (Germany) and 6.2% (Türkiye). In both countries, only few eyes were treated for ROP in zone III (Germany 1.4%, Türkiye 3.5%) (Figure 1C).

In the German cohort, treatment for the stage 3 eyes was more frequently initiated with fewer clock hours affected by neovascularizations compared to the Turkish cohort (one to three clock hours 23.5% vs. 16.6%). The majority of eyes in both countries, however, was treated with neovascularizations present in at least four clock hours (76.5% in Germany and 83.4% in Türkiye) (Figure 1D).

### ROP treatment

**FIGURE 2:**
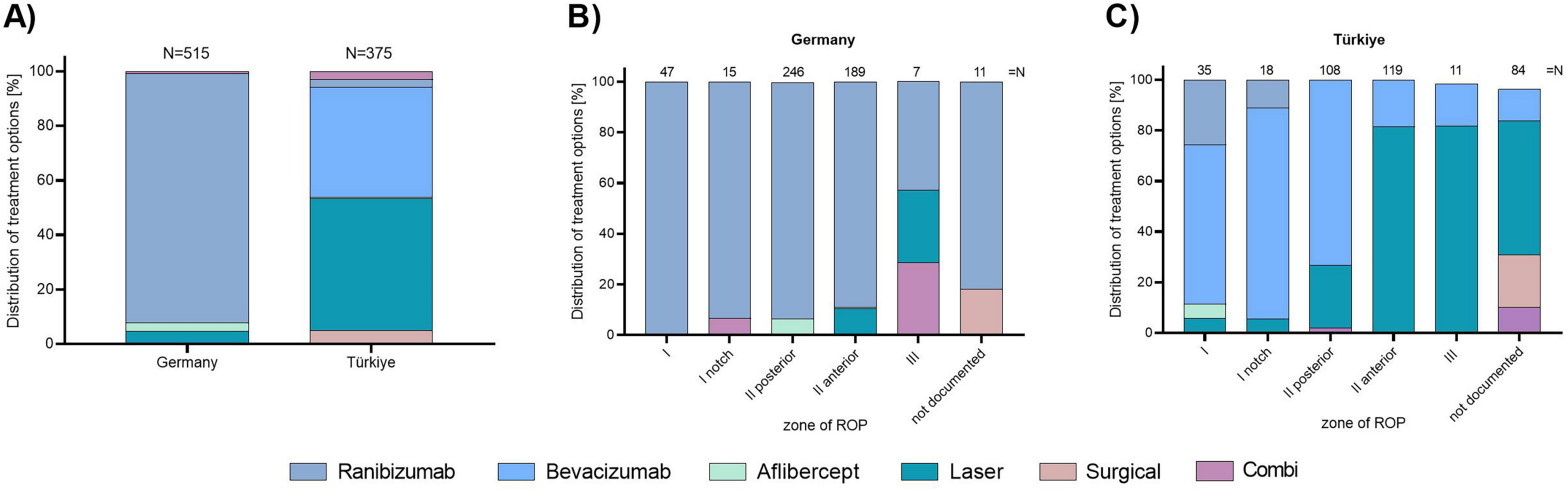
Initial treatment by country **(A**); initial treatment by zone in Germany **(B)** and Türkiye **(C)**. *N*, Eyes; surgical treatment: vitrectomy, scleral buckling, lensectomy or combinations of different surgical methods; Combi, combination: at least two treatment options combined (e.g., laser and anti-VEGF; surgical treatment and anti-VEGF; laser, anti-VEGF and vitrectomy).

More than 90% of infants in our cohort were treated bilaterally (93.3% in Germany; 90.9% in Türkiye). Treatment was applied in all but twelve of these infants in both eyes on the same day (only seven in Germany, five in Türkiye were initially treated bilaterally but on different days). The treatment method differed considerably between the two countries (Figure 2A). The vast majority of eyes at participating centers in Germany (94.4%) was treated with anti-VEGF (91.3% with ranibizumab, 3.1% with aflibercept), irrespective of the affected zone (Figure 2B). Laser coagulation was applied only to eyes treated for ROP in zone III and anterior zone II, in total only 4.3%. Four eyes (0.8%) received a combination of laser and ranibizumab. Two eyes (0.4%) in Germany were treated surgically (one eye with scleral buckling, and one eye with a combination of vitrectomy, lensectomy and synechiolysis) as initial treatment. In contrast, about half of all eyes at participating centers in Türkiye were treated with laser coagulation (48.2%). About 45% of eyes in Türkiye were treated with anti-VEGF, mainly bevacizumab (40.0%), and only a small percentage with ranibizumab (2.9%) and aflibercept (0.5%). As shown in Figure 2C, in Türkiye laser was mainly applied to eyes affected in more anterior zones (81.8% of eyes with zone III, 81.5% of eyes with zone II anterior). In contrast, in zone I and zone I secondary to notch, laser was used only in 5.7% and 5.6%, respectively and anti-VEGF was the treatment method of choice in most eyes with zone I (bevacizumab: 62.9%; ranibizumab: 25.7%; aflibercept 5.7%) and zone I secondary to notch (bevacizumab: 83.3%, ranibizumab: 11.1%). In the Turkish cohort, 4.8% of eyes were treated surgically as initial treatment (vitrectomy, scleral buckling, lensectomy, synechiolysis or a combination of different surgical treatment options) and 2.9% received a combination of different treatment methods (e.g., with a combination of laser, ranibizumab and vitrectomy; laser and bevacizumab; laser and ranibizumab; or bevacizumab, vitrectomy and lensectomy).

In the German cohort, we observed a very high frequency of anti-VEGF use throughout the whole observation period (2019–2025), with the first use of aflibercept documented in 2024 and increasing use in 2025. In the Turkish cohort, we observed an increase in the use of anti-VEGF (mainly bevacizumab) over our observation period: while in 2019 only 13.3% of eyes were treated with anti-VEGF, this rate increased to 56.3% in 2025) (Supplementary Figure 2).

Over half of all initial treatments in Germany (57.3%) were performed under analgosedation, followed by about a third (32.1%) under general anesthesia with intubation or laryngeal mask applied for ROP treatment. In an additional 6.9% of procedures, an existing intubation or laryngeal mask was already in place when ROP treatment was performed. Only 3.8% of infants were treated under local anesthesia in the German cohort. In Türkiye, more infants received intubation or laryngeal mask for ROP treatment (48.0%) and in an additional 13.6% an existing intubation or laryngeal mask was already in place when ROP treatment was performed. About a third (30.8%) of treatments in the Turkish cohort were performed under analgosedation, and 7.6% with local anesthesia alone (Supplementary Figure 3A). When analyzing the distribution of anesthesia by treatment modalities, we observed that in the Turkish cohort almost all laser coagulations were done under general anesthesia (96.8%), in the German cohort this was the case only in 70% while 30% were done under analgosedation, which was only used in 1.1% in the Turkish laser treated infants. The predominant form of anesthesia for anti-VEGF treatments was analgosedation in both countries (Germany 58.8% of ranibizumab treated eyes, Türkiye 75.6% of bevacizumab treated eyes). Some of the anti-VEGF treatments were done with local anesthesia alone (3.8% of infants treated with ranibizumab and 12.5% of infants treated with aflibercept in the German cohort, 14.1% of infants treated with bevacizumab and the one infant treated with aflibercept in the Turkish cohort), and in Türkiye even 2.1% of laser treatments were conducted under local anesthesia alone (Supplementary Figures 3B, C).

### ROP retreatment

In total, 894 eyes treated for ROP are included in this analysis. Of these, 23 eyes were initially treated with laser coagulation for PAR and not active ROP. In addition, one eye was treated surgically with a combination of a vitrectomy and a lensectomy due to persistent fetal vasculature. These 24 eyes were excluded from all analyses regarding ROP retreatment rates since their initial treatment was not for active treatment-warranted ROP. Therefore, 870 eyes are included in the analyses regarding retreatment rates. It should also be noted that retreatment can occur either because of reoccurrence of active ROP or for PAR, i.e., for stages that have no active treatment-warranted ROP.

**FIGURE 3:**
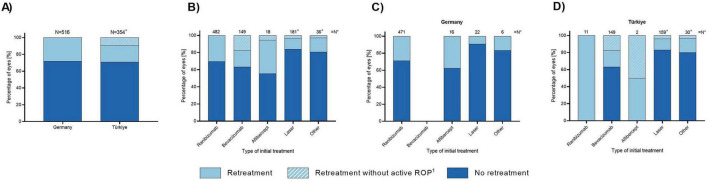
Retreatment rate by country **(A)**; retreatment rate by initial treatment method **(B)**; retreatment rate by initial treatment method in Germany **(C)** and Türkiye **(D)**. ^1^active ROP includes stage 5/ 4b/ 4a/ aggressive ROP (A-ROP)/ I,3+/ I,2+/ I,3–^#^/ I,1+^#^/ II,3+/ II,2+/ I,2–^#^/ II,1+^#^/ I,1–/ III,3+/ II,3–/ II,2–/ III,2+^#^/ II,1–/ III,1+^#^/ III,3–/ III,2–/ III,1–. The combinations marked with ^#^ were not present in this cohort. ^+^23 eyes initially treated with laser coagulation for PAR and not active ROP and 1 eye treated surgically due to persistent fetal vasculature are excluded from retreatment analyses. *For one eye in Germany and three eyes in Türkiye the type of initial treatment was not documented. These eyes are excluded in panels **(B–D)**.

A total of 248 eyes (28.5%) received at least one retreatment for ROP (28.1% in the German cohort and 29.1% in the Turkish cohort; Figure 3A). For demographic parameters at retreatment, see Table 1. Children in Germany had their first retreatment on average at an earlier postmenstrual age (PMA) and postnatal age (PNA) than infants in Türkiye and were also lighter at first retreatment. The time between initial treatment and first retreatment was significantly different between the two countries [around 50 days (range: 2–135 days) in Germany and 115 days (range: 1 to 396 days) in Türkiye; *p* < 0.0001].

When comparing retreatment rates by type of initial treatment, the highest retreatment rate was documented after aflibercept (44.4%; note: only 18 eyes were originally treated with aflibercept). The retreatment rate after bevacizumab was the second highest with 36.4% and the lowest retreatment rate of the three anti-VEGF agents was documented for ranibizumab with 30.5% (Figure 3B). Of the 181 eyes initially treated with laser coagulation (note 23 eyes were excluded here as they were initially treated only for avascular retina and not active ROP), 16.0% needed at least one additional treatment. Of the 34 eyes treated either surgically, or with a combination of different treatment methods as initial treatment, seven received an additional treatment (20.6%) (Figure 3B). The retreatment rates by initial treatment method differed between the two countries (Figures 3C, D): while all eleven eyes in Türkiye that had received ranibizumab as initial treatment received retreatment, less than a third of the eyes treated with ranibizumab as initial treatment in Germany received a retreatment (28.9%; 136 of 471 eyes). Retreatment rates after laser coagulation were higher in the Turkish cohort compared to the German cohort (17.0% vs. 9.1%). Here one has to keep in mind that especially in the Turkish cohort, anti-VEGF has been predominantly used in eyes with posterior ROP which might have affected the later need for retreatment (anti-VEGF was used in 94.3% of eyes affected with ROP in zone I; in 94.4% in zone I secondary to notch and in 73.1% in posterior zone II).

As pointed out above, it is important to distinguish between eyes that are retreated due to reactivation of treatment-warranted ROP and those that receive their retreatment due to PAR. Unfortunately, it is not possible to make this distinction with certainty based on the data entered into the EU-ROP database at the moment (a database amendment has just recently been implemented). In the German cohort, no eye was retreated with a documented stage of “no ROP,” but three eyes in Germany were retreated due to ROP stage 3- in zone III (one with ranibizumab, two with laser coagulation). In Türkiye, 34 eyes received retreatment with a documented stage of “no ROP” with laser coagulation. This was especially the case in the eyes initially treated with bevacizumab, where 47.3% of all retreatments (26 of 55) were performed at a documented stage of “no ROP”. In six of 27 eyes (22.2%) with retreatment after initial laser treatment, “no ROP” was documented at the time of retreatment. No eye initially treated with ranibizumab was retreated for “no ROP” (Figure 3D).

**FIGURE 4:**
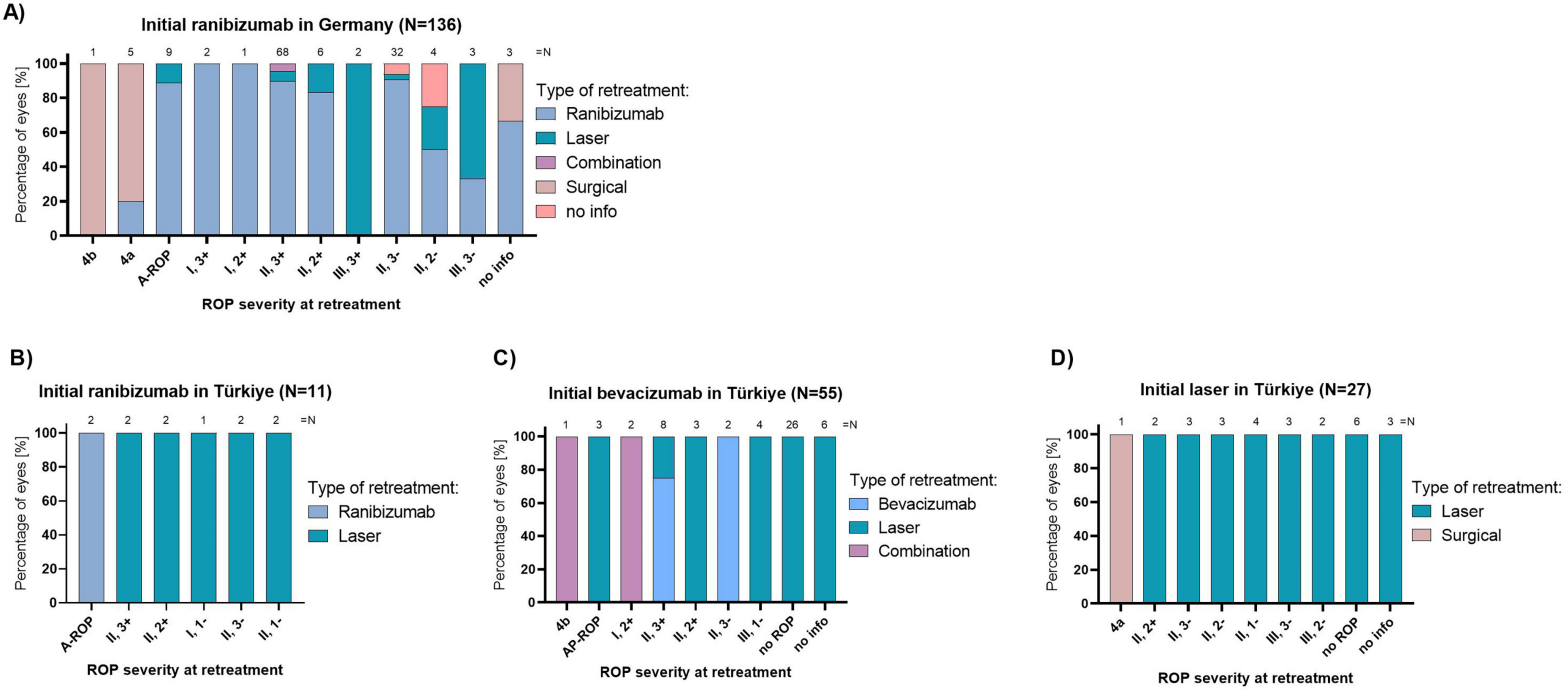
Type of retreatment by initial treatment method and ROP severity at retreatment per country. [**(A)** Germany initial ranibizumab; **(B)** Türkiye initial ranibizumab; **(C)** Türkiye initial bevacizumab; **(D)** Türkiye initial laser]. Nine further eyes were retreated in Germany: two after initial laser coagulation, retreatment with laser coagulation and cryocoagulation at II, 3+; one eye after an initial combination of laser and ranibizumab with a combination of laser and ranibizumab at I, 1–; six after initial aflibercept, retreatment of five eyes with aflibercept at II, 3+ and one eye with aflibercept at II, 3–. Ten further eyes were retreated in Türkiye: two for which type of initial treatment was not documented were treated with laser at unknow ROP severity; one after initial aflibercept with bevacizumab at II, 2– and one after initial aflibercept with laser at no ROP; six eyes after initial combination treatment: one surgical at 5 and one surgical at 4a, three surgical at I, 2+ and one with laser at no ROP.

The predominant treatment method for first retreatments in the German cohort was ranibizumab (76.6%) (Figure 4A), while in the Turkish cohort most eyes received laser coagulation as first retreatment (79.6%) (see Figures 4B–D). Overall, 55 eyes needed a second retreatment [29 of 516 in Germany (5.6%); 26 of 354 in Türkiye (7.3%)], 10 eyes required a third retreatment [nine in Germany (1.7%), six in Türkiye (1.7%)] and three eyes in Germany received even a fourth retreatment (0.6% of all eyes). Two eyes in Germany needed a fifth retreatment and one eye even six retreatments (see Supplementary Figures 4A,B for a detailed overview of the different types of retreatments).

### Unfavorable outcomes

The aim of ROP screening is to detect treatment-warranted ROP as early as possible to avoid progression to (partial) retinal detachment. At initial treatment, 20 eyes in the Turkish cohort were treated for a complete retinal detachment (ROP stage 5), three eyes (two in Germany, one in Türkiye) for stage 4b and eight eyes (three in Germany, five in Türkiye) for stage 4a (see Figure 1A and Supplementary Table 1). After initial treatment with ranibizumab for A-ROP, ROP stage 3+ in zone I, stage 3+ in zone II or stage 3- in zone II ten eyes of nine infants (2.1% of all eyes initially treated with ranibizumab) progressed to stage 4a, 4b or 5. One eye treated with bevacizumab for ROP stage 2- in zone I progressed to stage 4a (0.7% of all eyes initially treated with bevacizumab), one eye treated with laser for ROP stage 3- in zone II progressed to 4a (0.6% of all eyes initially treated with laser) and one eye that was initially treated with a combination of laser and bevacizumab for A-ROP progressed to stage 5 (see Supplementary Figure 5).

## Discussion

The EU-ROP registry was established in 2021 as an international expansion of the German Retina.net ROP registry. The objective of this registry is to study the typical clinical features of infants with treatment-warranted ROP and detect regional variations in phenotype, patient demographics and treatment patterns. Data collection in a joint database results in extensive comparable datasets forming the foundation for various analyses with comparisons on country level being one of them. It has to be kept in mind, that our data is not representative for Germany and Türkiye on a country level but only for participating centers. Referral patterns and center-specific treatment preferences may have influenced the reported distributions. While with 24 centers in Germany and eight centers in Türkiye, we do cover a significant portion of ROP treatment centers, values may be different at other centers not participating in the EU-ROP registry.

The key findings of this first comparison of two large contributors to the registry, Germany and Türkiye, are:

(1) demographical characteristics of infants treated for ROP differ considerably between the German and the Turkish cohort with infants in Türkiye being older at birth and at the time of initial treatment;

(2) while in Germany a large majority of eyes was treated for ROP stage 3+ in zone II, Türkiye had a higher variability in ROP severities at the time of initial treatment with more treatments both for incomplete vascularization as well as severe ROP (zone I and A-ROP) compared to Germany;

(3) over 90% of eyes at participating centers in Germany were treated with ranibizumab while in Türkiye laser coagulation and bevacizumab were the most prevalent treatment modalities with a significant treatment bias toward bevacizumab for more central (severe) ROP stages and laser therapy for more anterior (milder) stages;

(4) retreatment rate after ranibizumab was lower than after bevacizumab with two caveats: first, bevacizumab was mainly used (in Türkiye) for posterior ROP in a cohort of infants with smaller GA at birth and birth weight, while ranibizumab (in Germany) was also used in a high percentage of eyes with ROP in anterior zone II; second, about half (47.3%) of all retreatments after initial bevacizumab treatment were with laser treatment due to “no ROP” (PAR); retreatments after bevacizumab occurred considerably later compared to ranibizumab; laser therapy showed lower retreatment rates.

ROP is a disease that is highly sensitive to subtle differences in neonatal and ophthalmological care (2, 6). The neonatal mortality rate per 1,000 live births is twice as high in Türkiye (4.9 in 2023) than in Germany (2.3 in 2023), with an even higher difference between the two countries at the beginning of the observation period (5.6 in Türkiye vs. 2.3 in Germany in 2019) (7). Differences in neonatal intensive medicine may have influenced the observed differences in gestational age (GA) and birth weight of infants with treatment-warranted ROP between the German and Turkish cohort and may also have impacted the relatively higher rate of A-ROP in Türkiye (17.9%) in contrast to Germany (4.9%) (15). Also variations in oxygen management strategies between NICUs/countries may have contributed to the observed differences as oxygen exposure and fluctuations in oxygen saturation are well-established risk factors for the development of severe ROP (16). The method of oxygen supplementation is not documented in the EU-ROP registry, only the duration of oxygen supplementation (in days) until initial treatment, which did not differ significantly between the two groups (see Table 2). Interestingly, some reports from Türkiye found GA and birth weight data for Türkiye that were closer to those reported here for Germany (17) while others report almost identical values as reported in the current analysis (18). For Germany, the demographic characteristics at birth and at initial treatment are comparable to previous reports from the German Retina.net ROP registry and to other countries with highly developed neonatal care such as Sweden (19, 20). Countries like Poland and Greece, although their WHO neonatal mortality rate is comparable to Germany, have reported slightly higher GA (both 26 weeks) and birth weight among their infants treated for ROP (868 and 725 g) (21, 22). National ROP screening guidelines differ slightly between countries. According to the German screening guidelines, infants born at a gestational age (GA) of less than 31+0 weeks, or with a birth weight below 1,500 g if GA is unknown, are included in ROP screening (23). In Türkiye, infants born at ≤34 weeks GA or with a birth weight ≤ 1,700 g are screened (24). These national screening criteria are regularly revised and adapted based on epidemiological data and clinical experience to ensure that infants at risk for treatment-requiring ROP are appropriately identified and not missed. Therefore, we believe that the differences observed in our dataset are more likely to reflect the underlying clinical reality and disease burden within the respective populations rather than differences in guidelines.

While the majority of eyes in Germany were treated due to ROP stage 3+ in zone II (56.1%), this percentage was considerably lower than in previous studies of the German Retina.net ROP registry, where up to 76.6% had been diagnosed with ROP stage 3+ in zone II at initial treatment (19). The second most prevalent ROP severity in Germany was zone II, 3- (20.9%), of which 69.8% were diagnosed with pre-plus disease. This indicates that in many cases an earlier treatment is considered at German centers, potentially taking into account the ETROP study results indicating that in some cases earlier treatments can yield better results (25).

It is interesting to observe that at participating centers in the German cohort ranibizumab has almost completely replaced laser coagulation or bevacizumab treatment, irrespective of zone. This is different in the Turkish cohort where we observe a distinction between central ROP, which is primarily treated with anti-VEGF (bevacizumab predominating), and more peripheral ROP, where laser coagulation is more prevalent. The preference for bevacizumab observed in Türkiye may reflect the influence of national reimbursement policies on clinical decision-making. This finding underscores that the choice of anti-VEGF agent is not determined solely by clinical evidence but is also affected by country-specific healthcare policies and reimbursement frameworks. These contextual factors should be considered when interpreting international differences in treatment patterns. In both countries, aflibercept was documented in only very few cases (16 eyes in Germany, two eyes in Türkiye). Here one needs to keep in mind that aflibercept was only approved for ROP at the end of 2022 by the EMA and it will be interesting to observe how the use will evolve in the next years. In Germany we observed an increase in the use of aflibercept from 2024 (3.7%) to 2025 (22.6%). For Türkiye, only two eyes were treated in 2022 with aflibercept in the current cohort. For Türkiye, however, several reports have been published on the use of aflibercept for ROP (26–28). Unfortunately, not all Turkish centers are participating in the EU-ROP registry, a limitation that we strive to overcome in the coming years.

One possible reason why laser treatment may be preferred over anti-VEGF in Turkish centers may be the fact that treatment centers in Türkiye are often referral centers and infants and their families may live far away from the treatment center, rendering frequent follow-ups, as required after anti-VEGF, difficult. In these cases, a definitive treatment with laser coagulation might be preferred (17, 29).

The high percentage of ranibizumab use in Germany (more than 90% at participating centers) is striking, even compared to other Western countries like Sweden where in 2020 and 2021 anti-VEGFs in total were only used in less than 30% of children (30), Poland (data from 2021, about 35% ranibizumab) (21) or the US (2015–2017 about 30% anti-VEGF, no newer data available) (31).

Interestingly, despite considerable differences in treatment modalities between Germany and Türkiye, the retreatment rates seem comparable (28.1% in the German cohort vs. 29.1% in the Turkish one). It is, however, very important to differentiate in this context, why a retreatment was performed. Unfortunately, the reason for retreatment is currently not a specific question in the EU-ROP questionnaire. This has just recently been changed with a database amendment in October 2025 and will thus be available in future analyses. For the study population investigated here we can, however, only speculate that the severity of ROP at the time of retreatment may reflect to some degree whether active treatment-warranted ROP or PAR were the reasons why retreatment was performed. While in Germany all eyes at the time of retreatment were documented with some severity of ROP (although it is debatable if ROP III 3- actually reflects a treatment-warranted ROP stage or may rather be treatment for PAR), in Türkiye we observed 33.0% of eyes at retreatment without any stage of active ROP (i.e., but avascular peripheral retina at time of retreatment). No ROP, when excluding all eyes with “no ROP” at time of retreatment, the overall retreatment rate in Türkiye is reduced from 29.1% to 19.5%. In addition, the considerably higher mean PMA at retreatment in Türkiye compared to Germany (57.8 vs. 42.1 weeks) may serve as an indicator that a certain percentage of eyes in Türkiye received their second treatment for PAR and not active ROP. Differences in follow-up accessibility and referral geography may influence retreatment strategies, especially regarding prophylactic laser treatment for PAR after anti-VEGF therapy. Currently, there is no global consensus on the management of PAR, and the necessity of laser intervention in these quiescent cases remains a subject of debate, but retreatment indications appear to be fundamentally different between the two observed cohorts.

As mentioned before, one of the limitations of the EU-ROP registry currently still is that the data are only representative for participating sites and not the whole countries. We strive to overcome this limitation in the next years as we are continuously working on expanding the registry within countries and also to additional countries in geographical Europe. Another limitation of this study is that no multivariate analysis was performed to adjust for potential differences in demographic parameters between countries. Therefore, the observed differences should be interpreted with caution, as residual confounding factors cannot be excluded. Given the exploratory and descriptive nature of the study, we focused on reporting unadjusted comparisons. While in general the ICROP3 classification system was used for grading ROP severity, we do not collect information on possible grading specificities at participating centers. Center-specific variables, for example in grading A-ROP or other ROP stages, may therefore affect ROP severity gradings. Across the relatively large number of centers in both countries, however, we feel that center-specific confounders should be limited when interpreting the aggregated data per country.

## Conclusion

In summary, this is the first analysis from the EU-ROP registry comparing real-world treatment data between two countries. We observed considerable differences between the Turkish and German cohorts regarding demographic data, ROP severities, treatment patterns, and retreatment approaches. With more data from more centers across Europe to come, we hope to continue to contribute to a better understanding of ROP treatment and outcome. In this manuscript we still lack long-term structural or visual outcomes, which would be of particular interest given the differences in anti-VEGF and laser treatment strategies in the two countries. These parameters are collected in the EU-ROP registry, but can usually only reliably be assessed after the treated children reach a certain age. For more details, please visit www.eu-rop.org.

## Data Availability

The datasets presented in this article are not readily available because of data protection reasons. Requests to access the datasets should be directed to johanna.pfeil@med.uni-greifswald.de

## EU-ROP Study Group

Austria: **University Hospital Salzburg:** Jakob Briem, Johann Colvin, Stefan Egger, Herbert Reitsamer, Patrick Schernthaner, Sandra Stuppner, Eva Wiener. Bulgaria: **Acibadem City Clinic Tokuda University Hospital EAD:** Lilyana Petrova Dimitrova, Iliyana Georgieva Ilieva, Gergana Svetoslavova. **University Hospital Alexandrovska:** Ognyan Mitkov Mladenov, Alexander Hugo Oscar. **University Hospital Saint George, University Eye clinic and Medical University:** Yanitsa Kondova-Atanassova, Vasil Marinov. Croatia: **Clinical Hospital Sveti Duh:** Mirjana Bjelos Roncevic, Jelena Doresic, Edita Kondza Krstonijevic, Rebeka Ribičić. Czech Republic: **České Budějovice:** Jiří Dušek, David Honer, Michaela Štěpanyová, Barbora Vařejková. Estonia: **Eye Clinic of Tartu University Hospital:** Piret Jüri, Liina Kala, Mikk Pauklin. France: **Hospital Rothschild Paris:** Amandine Barjol, Georges Caputo, Fiammetta Catania, Florence Metge-Galatoire. Germany: **Carl-Thiem University Hospital Cottbus:** L. Geerdts, Madlen Löbel, Lisa Starke. **City Hospital Braunschweig:** Erik Chankiewitz, Spyridon Monastiriotis, Georgia Potaki. **Clinic Stuttgart Location Katharinenhospital, part of Olga Hospital:** Florian Gekeler, René Machulla, Marius Metz. **Ernst von Bergmann Hospital Potsdam:** Ameli Gabel-Pfisterer, Thomas Pohlenz, Julia M. Pulst Caliman, Sophie Korn, Sandra Kroll, Martina Möglich. **Helios Hospital Berlin-Buch:** Helge Breuß, Anne-Kathrin Retzlaff. **Jendritza Ophthalmic Practice, Ludwigshafen**: Waldemar Jendritza, Bettina Jendritza. **Nürnberg Clinic South:** Zakaria Drebi, Florian Schindler. **St. Franziskus Hospital Münster:** Henrik Faatz, Leonie Kläver, Andrea Koschinski, Denise Ruch, Georg Spital. **University Hospital Bonn:** Michael Petrak, Kristina Pfau, Marlene Saßmannshausen. **University Hospital Chemnitz:** Annika Ader, Katrin Engelmann, Ulrike Fleischer, Imke Goldammer, Vinodh Kakkassery, Grabiela Krestanova, Franziska Mühmel, Pavel Smetana, Martin Tiez, Anja Wienigk. **University Hospital Düsseldorf:** Jemina Benga, Rainer Guthoff, Stephan Jansen, Sema Kaya, Ala Khamees, Jennifer Prues-Hölscher, Alexandra Schilcher, Verena Schöneberg. **University Hospital Erlangen:** Alexander Bartsch, Lisa Dussing, Sabrina Frank, Karolina Motloch. **University Hospital Essen:** Eva Biewald, Tobias Kiefer, Mael Lever, Philipp Rating. **University Hospital Freiburg:** Tim Bleul, Alexandra T. Camp, Navid Farassat, Nikolai Gross, Lutz Joachimsen, Sebastian Küchlin, Wolf A. Lagrèze, Luca Malagutti, Fanni E. Molnár, Michael Reich, Anne Schwietering. **University Hospital Fulda:** Iva-Ruth Bartmann, Fithri Indraswari. **University Hospital Gießen:** Monika Andrassi-Darida, Kerstin Holve, Kafa Ilia, Lyubomyr Lytvynchuk. **University Hospital Göttingen:** Tarek Aljarmakani, Sebastian Bemme, Jana Katharina Dieks, Mohammed Khattab, Peer Lauermann, Yulia Meyer. **University Hospital Greifswald:** Hagen Bahlmann, Matthias Heckmann, Knud Linnemann, Konrad Schulze, Andreas Stahl, Allam Tayar. **University Hospital Hamburg-Eppendorf:** Hanna Faber, Nefeli Kounatidou, Caroline Lago, Laurenz Johannes Bernhard Pauleikhoff, Tim Schade, Christos Skevas, Martin Spitzer, Annabelle Volk, Jan Wildner, Denise Yang-Seeger. **University Hospital Hannover:** Anna Bajor, Anke Beckmann, Carolin Böhne, Bettina Bohnhorst, Dorothee Brockmann, Meltem Elcivan, Carsten Framme, Thomas Gröber, Melanie Haar, Jonas Herden, Karsten Hufendiek, Christoph Jacobi, Christina Jacobsen, Nils Mester, Corinna Peter, Amelie Pielen, Sabine Pirr, Tjark Rauscher, Alena Riesmeier, Lorena Stachowski, Nora Wojtera, Aida Shaghaghi Zadeh. **University Hospital Köln:** Tim U. Krohne, Jeany Li. **University Hospital Mannheim:** Nour Arbaoui, Sabine Buchner, Felix Dittgen, Elke Edelmann, Robert P. Finger, Mustafa Kamal Hallak, F. Kipfmüller, Sina Koch, Jonathan Schaeff, Sylvia Selig. **University Hospital Münster:** Verena Englmaier, Nicole Eter, Silvia Falkenau, Barbara Glitz, Sarah Kleemann, Camila Matias, Jenny Potratz, Julia Sandkötter, Jens Storp, Maria Tekaat, Cedric Weich. **University Hospital Regensburg:** Teresa Barth, Lauretta Gluth, Horst Helbig, Herbert Jägle Annette Keller-Wackerbauer, Florian Langhammer, Holger Michel, Sven Wellmann. **University Hospital Rostock:** Steffi Knappe. **University Hospital RWTH Aachen:** Sabine Baumgarten, Volker Buck, Emilia Michalewicz, Peter Walter, Tibor Lohmann. **University Hospital Schleswig-Holstein:** Viktorija Belousova, Maximilian Busch, Stefanie Gniesmer, Salvatore Grisanti, Julius Caspar Rotering, Christian Schiemenz, Philip Schubart. **University Hospital Tübingen:** Axel Frank, Ulrike Hagemann, Benedikt Rössler, Daniela Süsskind, Michael Völker. **University Hospital Ulm:** Britta Dahlke, Melih Parlak, Ingrid Weithmann, Armin Wolf, Johannes Kahle, Wessam Alkabouni. **Vivantes Hospital Neukölln:** Sabine Aisenbrey, Lisa Fu, Marian Liegl, Melanie Michelczak. Greece: **Papageorgiou General Hospital of Thessaloniki, Aristotle University of Thessaloniki:** Maria Lithoxopoulou, Asimina Mataftsi, Stella Moutzouri, Christos Tsakalidis, Nikolaos Ziakas. **P&A Kyriakou Athens Children Hospital:** Theodoros Gkouvas, Tataropoulou Kassandra, Agathi Kouri, Anna Mourgela, Angeliki Nika, Margarita Papadopoulou. **University Hospital of Larissa:** Sofia Androudi, Maria Goudesidou, Ioanna Grivea, Katerina Kaffe, Eleni Papageorgiou, Zoi Tsani. Hungary: **Semmelweis University:** Teodóra Dusek, Erika Maka, Miklós Szabó, Andrea Szigeti, Lilána Szücs-Székács. Kosovo: **University Clinical Center of Kosovo:** Mediha Bahtiri, Fjolla Bokshi, Shukrije Brajshori, Florie Greicevci, Luan Morina, Belinda Pustina, Lisa Qurdina, Ardian Shabani. Moldova: **Gheorghe Paladi Hospital:** Mazur Liubovi. Poland: **Prof. Dr. Stanislaw Popowski Regional Specialized Children’s Hospital:** Maria Szwajkowska. **Children’s Memorial Health Institution:** Kristof Cieslik, Agnieszka Czeszyk, Dariusz Gruszfeld, Wojciech Hautz, Beata Kocyla-Karczmarewicz, Klaudia Rakusiewicz-Krasnodȩbska, Anna Rogowska. **Medical University of Bialystok Children’s Clinical Hospital:** Monika Oziȩbło-Kupczyk, Joanna Zawistowska. **University Hospital in Bydgoszcz:** Magdalena Kaszuba-Modrzejewska. **University Hospital in Zabrze Silesia:** Bogumiła Wójcik-Niklewska. **University of Medical Sciences Poznan:** Elżbieta Czapla, Julia Dezor-Garus, Anna Gotz-Wiȩckowska, Jolanta Kaczmarek, Jan Mazela, Marta Pawlak, Tomasz Szczapa, Dawid Szpecht. Romania: **Cluj Country clinical emergency hospital:** Nicoleta Grosu, Mădălina Hapca, Monica Hăşmăşanu, Melinda Matyas, Simona Nicoară, Claudia ştefănuţ, Gabriela Zaharie. Spain: **Biomédica de Málaga y Plataforma en Nanomedicina:** Julia Escudero Gómez. **University and Polytechnic Hospital La Fe:** Lorena Azorín-Pérez, Honorio Barranco-González, Carlos Cauto-Picazo, Isabel Pascual-Camps, Marta Solaz-Ruiz, Ester Torres-Martínez. Switzerland: **Service d’ophtalmologie University Hospital, Geneva:** Franscisca Barcos Munoz, Walid Bouthour, Eline De Clerck, Gunda Kann, Mateusz Kecik, Martina Kropp, Ariane Malclès, Gabriele Thumann, Tin Vo. **University Hospital Basel:** Josep Callizo Planas, Daniela Hauenstein, Jordan Löliger. Türkiye: **Ankara University:** Huban Atilla, Pınar Bingöl Kızıltunç, Feyza Çalış Karanfil, Emel Okulu, Bensu Sezer. **Baskent University:** Imren Akkoyun, Ayse Ecevit, Gülsah Gökgöz, Deniz Anuk Ince, Gursel Yilmaz. Ebru Esen for the center of **Cukurova University:** Puren İsik, Hacer Yapicioglu Yildizdas. **Erciyes University, Faculty of Medicine:** Cagatay Karaca, Mehmet Mutlu, Osman Ahmet Polat, Huseyin Pur. **Etlik Zübeyde Hanim Women’s Health Education and Research Hospital:** Ahmet Yağmur Baş, Caner Kara, Ayşen Sumru Kavurt, Betül Ortatatlı. **Gazi University:** İbrahim Murat Hirfanoğlu, Benay Karabulut, Esin Koç, Şengül Özdek, Hüseyin Baran Özdemir. **Hacettepe University Children’s Hospital:** Figen Bezci Aygün, Tolga Çelik, İrem İyigün, Sibel Kadayıfçılar. **Ondokuz Mayis University:** Ozlem Eski Yucel. **Ordu University Medical Faculty:** Aslihan Uzun. **Uludag University Faculty of Medicine:** Nilgün Köksal, Meral Yıldız. **Umraniye Research and Education:** Ilke Mungan Akin. Ukraine: **Ivano-Frankivsk Oblast Dytyacha Klinichna Likarnya:** Tetiana Havrylyshyn, Paul Runge. **Kiev pediatric Hospital Ohmatdyt:** Sergiy Lupyr. **The Filatov Institute of Eye Diseases and Tissue Therapy of NAMS of Ukraine:** Serdeha Olha Oleksandrivna, Katsan Sergii Volodymyrovych.
