# Supplementary material for: Infants with retinopathy of prematurity (ROP) in Germany and Türkiye–a comparison based on data from the EU-ROP registry
Source: Front Med (Lausanne). 2026 Jul 29;13:1868323. doi: 10.3389/fmed.2026.1868323 (PMC13463155; doi:10.3389/fmed.2026.1868323)

Supplementary figure 1. Flow chart of patients in this analysis.

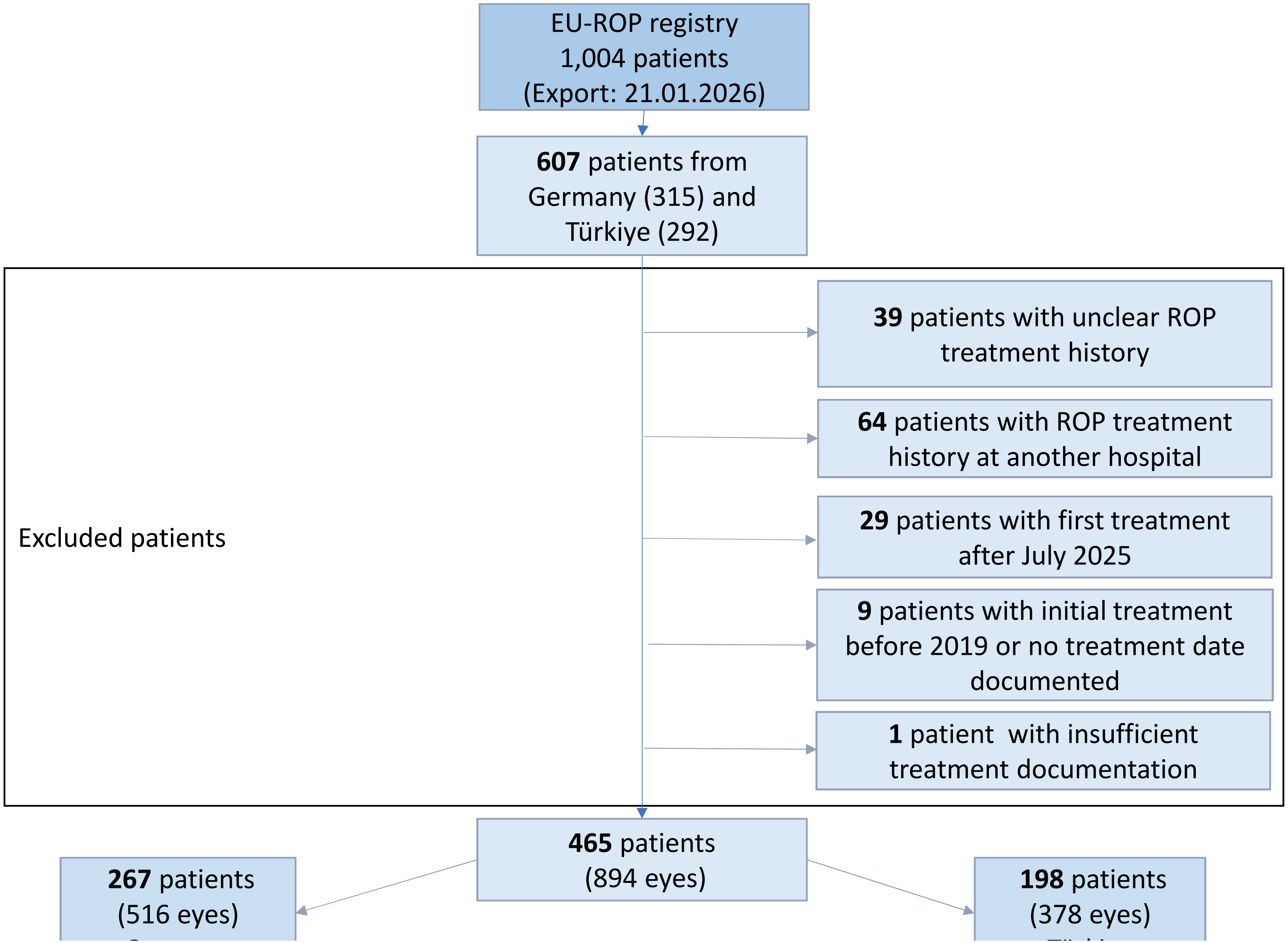

Supplement: Supplementary file 1 [file Image_1.pdf]
