# Supplementary material for: Infants with retinopathy of prematurity (ROP) in Germany and Türkiye–a comparison based on data from the EU-ROP registry
Source: Front Med (Lausanne). 2026 Jul 29;13:1868323. doi: 10.3389/fmed.2026.1868323 (PMC13463155; doi:10.3389/fmed.2026.1868323)
Supplement: Supplementary file 3 [file Image_3.pdf]

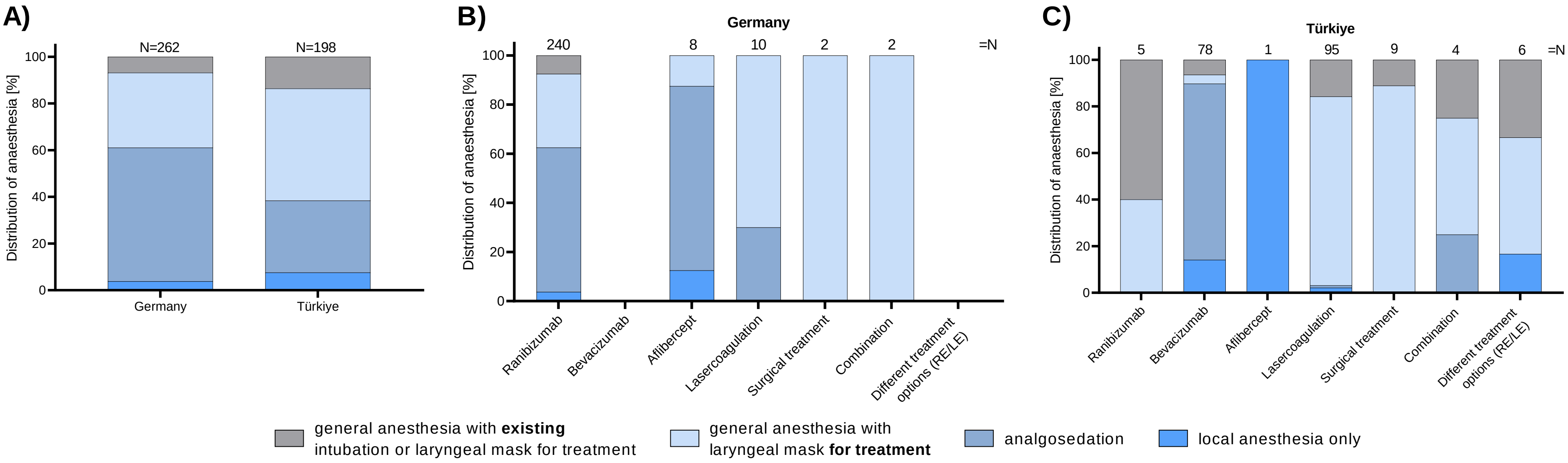
A)
N=262
N=198
Distribution of anaesthesia [%]
0
20
40
60
80
100
Germany
Türkiye
B)
Germany
240
8
10
2
2
=N
Distribution of anaesthesia [%]
Ranibizumab
Bevacizumab
Aflibercept
Lasercoagulation
Surgical treatment
Combination
Different treatment options (RE/LE)
C)
Türkiye
5
78
1
95
9
4
6
=N
Distribution of anaesthesia [%]
Ranibizumab
Bevacizumab
Aflibercept
Lasercoagulation
Surgical treatment
Combination
Different treatment options (RE/LE)
general anesthesia with existing intubation or laryngeal mask for treatment
general anesthesia with laryngeal mask for treatment
analgosedation
local anesthesia only
