# Supplementary material for: Infants with retinopathy of prematurity (ROP) in Germany and Türkiye–a comparison based on data from the EU-ROP registry
Source: Front Med (Lausanne). 2026 Jul 29;13:1868323. doi: 10.3389/fmed.2026.1868323 (PMC13463155; doi:10.3389/fmed.2026.1868323)
Supplement: Supplementary file 4 [file Image_4.pdf]

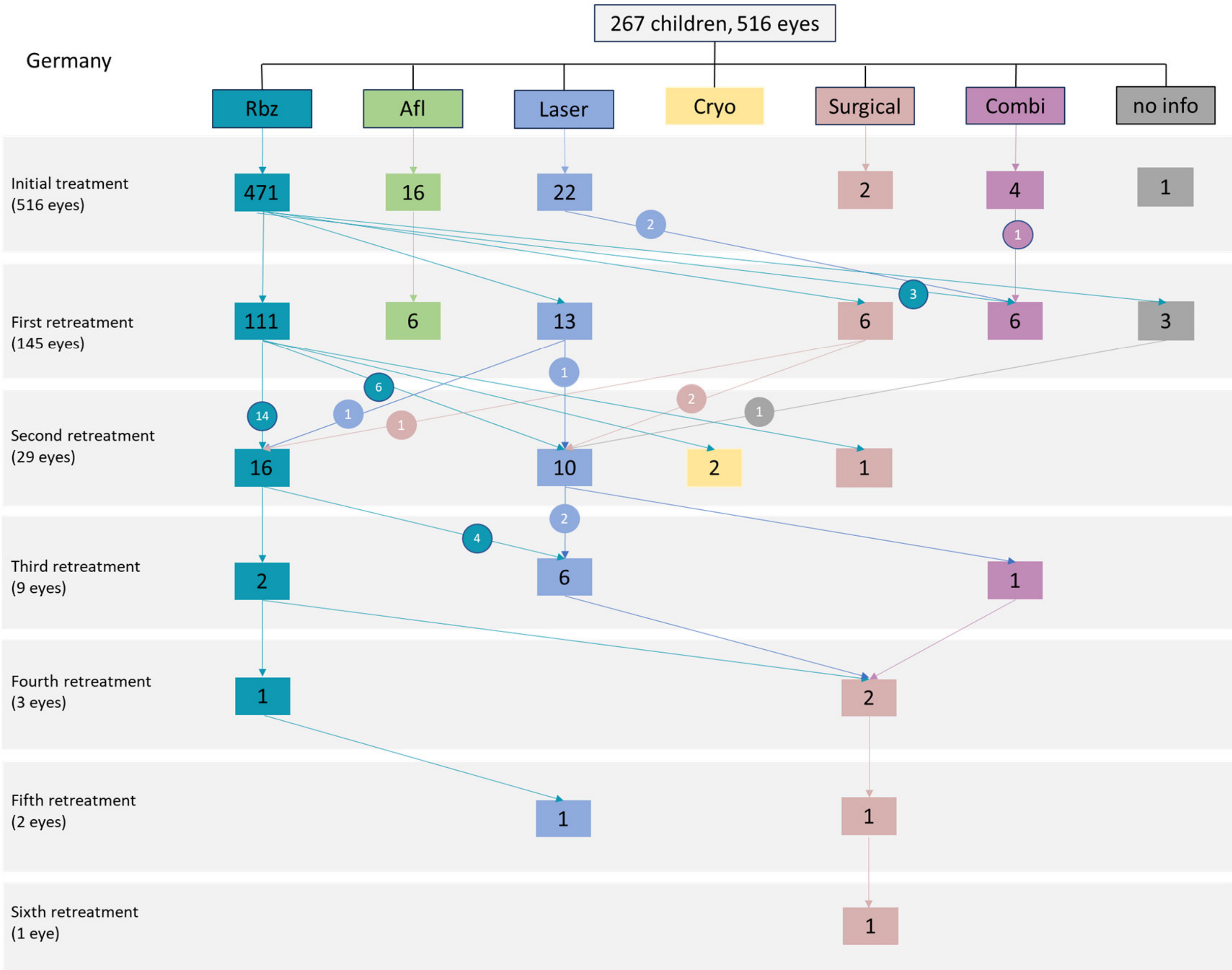

Supplementary figure 4A. Retreatment method by initial treatment method in Germany

Rbz=Ranibizumab; Afl=Aflibercept; Cryo=Cryocoagulation; Surgical: vitrectomy, scleral buckling, lensectomy or combinations of different surgical methods

Combi=combination: at least two treatment options combined (e.g. laser and anti-VEGF; surgical treatment and anti-VEGF; laser, anti-VEGF and vitrectomy)
