# Supplementary material for: Infants with retinopathy of prematurity (ROP) in Germany and Türkiye–a comparison based on data from the EU-ROP registry
Source: Front Med (Lausanne). 2026 Jul 29;13:1868323. doi: 10.3389/fmed.2026.1868323 (PMC13463155; doi:10.3389/fmed.2026.1868323)
Supplement: Supplementary file 5 [file Image_5.pdf]

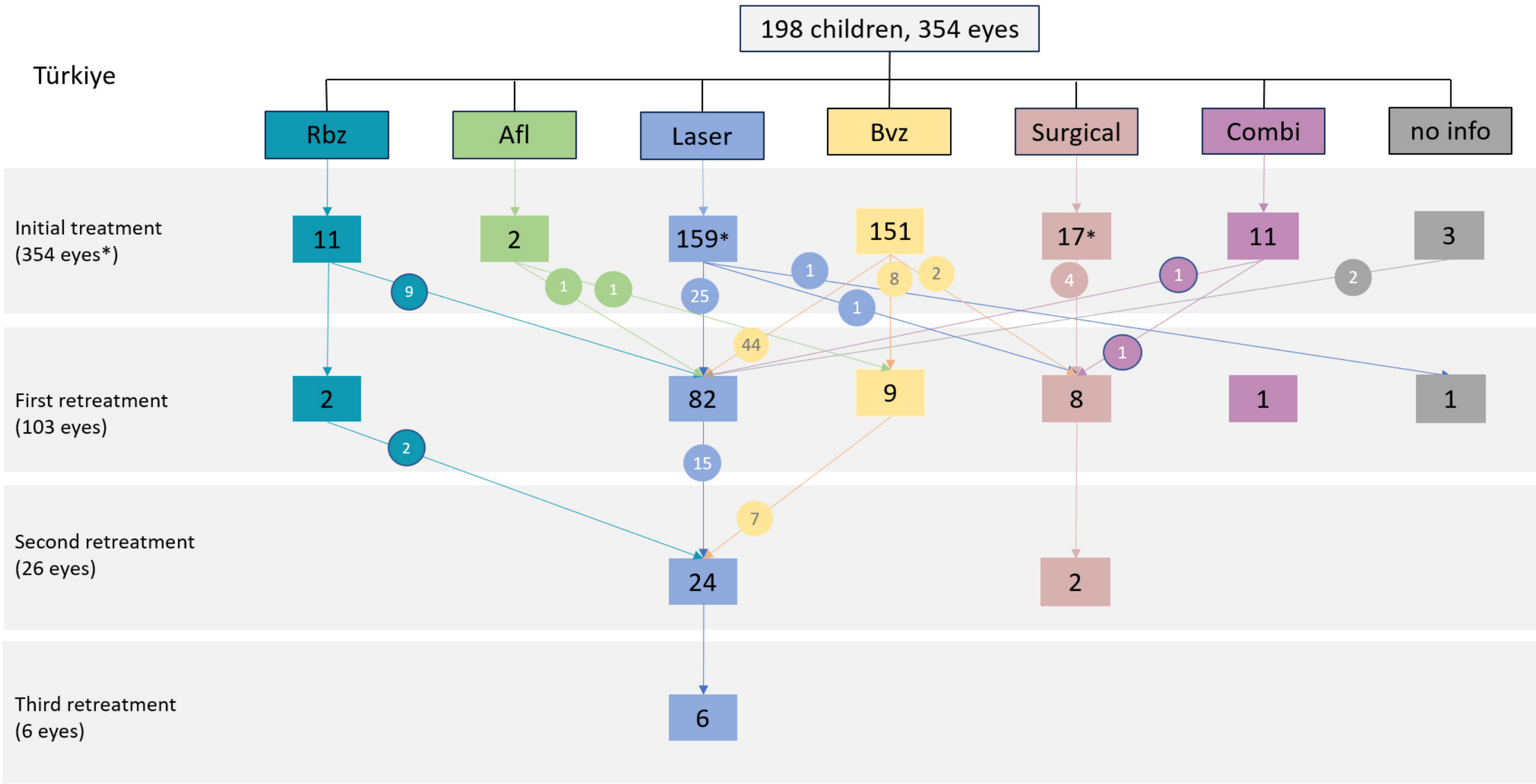

Supplementary figure 4B. Retreatment method by initial treatment method in Türkiye
Rbz=Ranibizumab; Afl=Aflibercept; Bvz=Bevacizumab; Surgical: vitrectomy, scleral buckling, lensectomy or combinations of different surgical methods
Combi=combination: at least two treatment options combined (e.g. laser and anti-VEGF; surgical treatment and anti-VEGF; laser, anti-VEGF and vitrectomy); *23 eyes initially treated with laser coagulation and 1 eye treated surgically due to an incomplete vascularization are excluded from retreatment analyses.
