# Supplementary material for: Infants with retinopathy of prematurity (ROP) in Germany and Türkiye–a comparison based on data from the EU-ROP registry
Source: Front Med (Lausanne). 2026 Jul 29;13:1868323. doi: 10.3389/fmed.2026.1868323 (PMC13463155; doi:10.3389/fmed.2026.1868323)
Supplement: Supplementary file 6 [file Image_6.pdf]

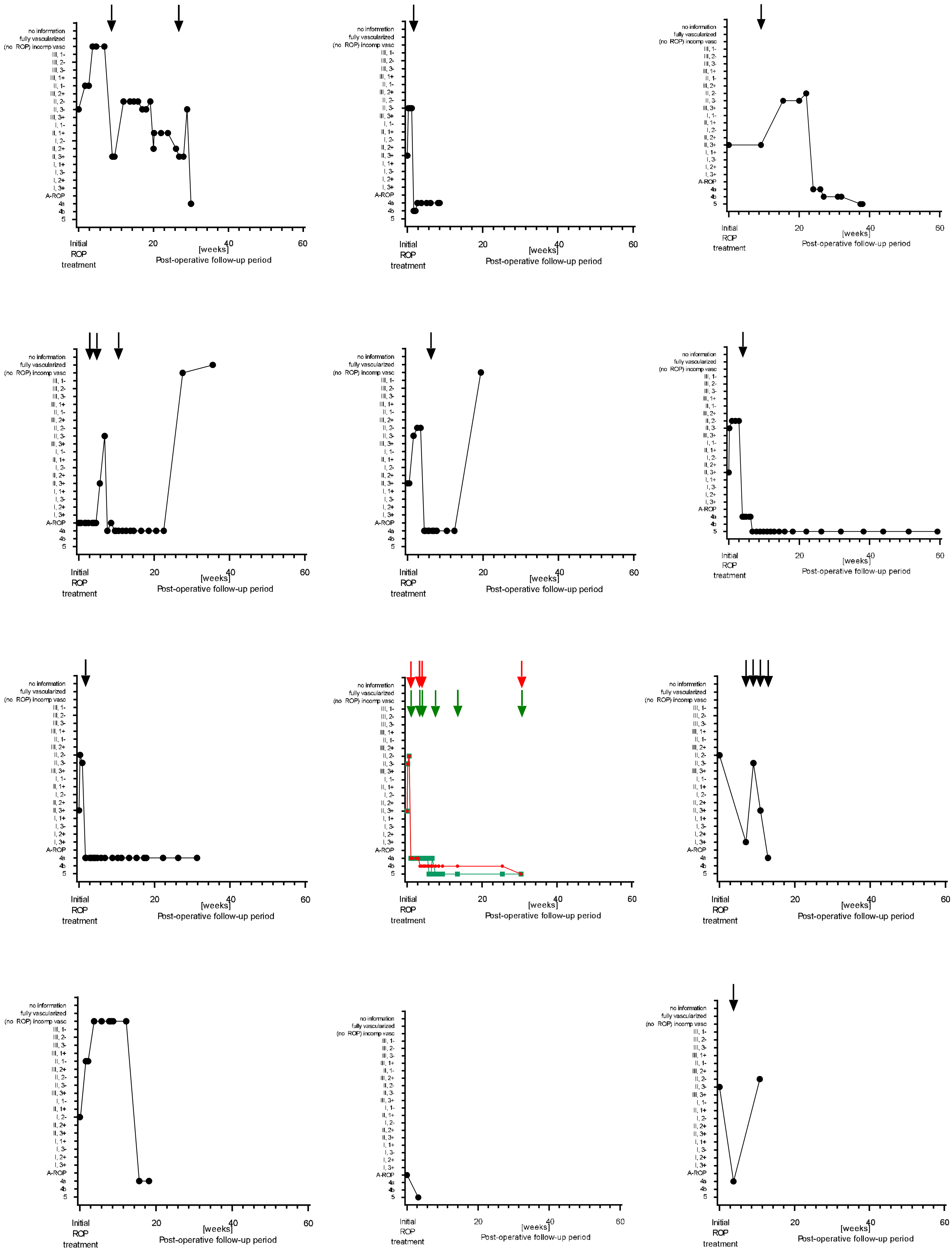

Supplementary figure 5. Development of ROP severity in eyes that progress to stage 4a, 4b, or 5 during postoperative follow-up.
Each graph represents one child. Only one child progressed to stage 5 in both eyes. Arrows above in the corresponding colors mark retreatments of the respective eye. Symbols indicate the time of follow-up examination and the corresponding ROP severity.
