# Supplementary material for: Infants with retinopathy of prematurity (ROP) in Germany and Türkiye–a comparison based on data from the EU-ROP registry
Source: Front Med (Lausanne). 2026 Jul 29;13:1868323. doi: 10.3389/fmed.2026.1868323 (PMC13463155; doi:10.3389/fmed.2026.1868323)
Supplement: Supplementary file 7 [file Table_1.docx]

| **ROP severity** | **Germany**  **(N=508 eyes)** | **Türkiye**  **(N=341 eyes)** | **Total**  **(N=849 eyes)** |
| --- | --- | --- | --- |
| 5 | 0 (0.0) | 20 (5.9) | 20 |
| 4b | 2 (0.4) | 1 (0.3) | 3 |
| 4a | 3 (0.6) | 5 (1.5) | 8 |
| A-ROP | 25 (4.9) | 61 (17.9) | 86 |
| I, 3+ | 25 (4.9) | 10 (2.9) | 35 |
| I, 2+ | 6 (1.2) | 3 (0.9) | 9 |
| I, 3- | 12 (2.4) | 1 (0.3) | 13 |
| I, 1+ | 2 (0.4) | 0 (0.0) | 2 |
| II, 3+ | 285 (56.1) | 75 (22.0) | 360 |
| II, 2+ | 21 (4.1) | 35 (10.3) | 56 |
| I, 2- | 1 (0.2) | 2 (0.6) | 3 |
| II, 1+ | 0 (0.0) | 0 (0.0) | 0 |
| I, 1- | 0 (0.0) | 0 (0.0) | 0 |
| III, 3+ | 6 (1.2) | 0 (0.0) | 6 |
| II, 3- | 106 (20.9) | 59 (17.3) | 165 |
| II, 2- | 10 (2.0) | 33 (9.7) | 43 |
| III, 2+ | 0 (0.0) | 0 (0.0) | 0 |
| II, 1- | 3 (0.6) | 1 (0.3) | 4 |
| III, 1+ | 0 (0.0) | 0 (0.0) | 0 |
| III, 3- | 0 (0.0) | 5 (1.5) | 5 |
| III, 2- | 0 (0.0) | 6 (1.8) | 6 |
| III, 1- | 1 (0.2) | 0 (0.0) | 1 |
| No ROP (incomplete vascularization) | 0 (0.0) | 23 (6.7) | 23 |
| Fully vascularized | 0 (0.0) | 1 (0.3) | 1 |

**Supplementary table 1.** Severity of ROP according to the modified ROP severity score.(13) For 8 eyes in Germany and for 14 eyes in Türkiye ROP severity was (partly) not documented (e.g. stage, zone or information on plus disease missing).
